# Dispersal depends on body condition and predation risk in the semi-aquatic insect, *Notonecta undulata*

**DOI:** 10.1002/ece3.1508

**Published:** 2015-05-20

**Authors:** Celina B Baines, Shannon J McCauley, Locke Rowe

**Affiliations:** 1 Department of Ecology & Evolutionary Biology, University of Toronto25 Willcocks St., Toronto, Ontario, M5S3B2, Canada; 2 Department of Biology, University of Toronto Mississauga3359 Mississauga Road North, Mississauga, Ontario, L5L1C6, Canada

**Keywords:** Body condition, dispersal, *Notonecta*, predation risk

## Abstract

Dispersal is the movement of organisms across space, which has important implications for ecological and evolutionary processes, including community composition and gene flow. Previous studies have demonstrated that dispersal is influenced by body condition; however, few studies have been able to separate the effects of body condition from correlated variables such as body size. Moreover, the results of these studies have been inconsistent with respect to the direction of the relationship between condition and dispersal. We examined whether body condition influences dispersal in backswimmers (*Notonecta undulata*). We also tested whether an interaction between body condition and predation risk (another proximate factor that influences dispersal) could contribute to the previously observed inconsistent relationship between condition and dispersal. We imposed diet treatments on backswimmers in the laboratory, and measured the effects of food availability on body condition and dispersal in the field. We found that dispersal was a positive function of body condition, which may have important consequences for population characteristics such as the rate of gene flow and population growth. However, the effects of body condition and predation risk were additive, not interactive, and therefore, our data do not support the hypothesis that the interaction between condition and predation risk contributes to the inconsistency in the results of previous condition-dependent dispersal studies.

## Introduction

Dispersal is the movement of organisms across space from a natal site or site of reproduction to a new site of reproduction (Howard 1960; Matthysen 2012). Therefore, dispersal encompasses both the movement of individuals across physical space, as well as the potential for gene flow among populations. Both of these aspects of dispersal can have important implications for ecological and evolutionary processes including metapopulation synchrony and persistence (Kuno 1981; Pulliam 1988; Ranta et al. 1995; Kendall et al. 2000), local and regional species composition (Clobert et al. 2012), and local adaptation (Holt and Gomulkiewicz 1997; Wade and Goodnight 1998; Rasanen and Hendry 2008).

Most investigations of the ecological and evolutionary consequences of dispersal have treated dispersal as a population-level characteristic, with each population having a single dispersal rate (e.g., Pulliam 1988). In contrast, there was an early recognition that dispersal propensity varies among individuals within a population (Howard 1960), and evidence that dispersal capacity and propensity depend on numerous phenotypic traits (O'Riain et al. 1996; Meylan et al. 2002; Bowler and Benton 2009). If dispersal sorts individuals across space according to phenotype, this has important implications for ecological and evolutionary processes, an idea that is already present in the literature in various forms. For example, Stamps (2006) proposed that high-condition dispersers are able to sample more habitats and are therefore more likely to settle in high-quality patches. This process sorts individuals into patches based on condition, adding to inherent differences between low- and high-quality patches that influence population growth rates, and possibly affect metapopulation dynamics. Shine et al. (2011) suggested that dispersal allows individuals to choose habitat patches that match their phenotype, thereby forcing assortative mating and driving evolution that mimics the process of local adaptation. The first step toward understanding these potential consequences of nonrandom dispersal is to characterize how phenotypic variation underlies variation in dispersal.

“Body condition” is a general term that may refer to any of several traits indicative of the overall health or performance potential of an animal, including energy reserves. Body condition in nature is often correlated with a number of other variables including maternal effects, the quality of the home patch, structural body size, and behavioral characteristics such as aggressiveness (Clobert et al. 2001; Benard and McCauley 2008). Most previous studies of condition-dependent dispersal have not been able to separate the effects of body condition from these correlates (e.g., O'Riain et al. 1996; Remy et al. 2011). One goal of this study was to manipulate body condition, or energy reserves, so that it was uncorrelated with other factors which might also influence dispersal, to determine whether body condition directly affects dispersal rates.

Empirical studies of the link between body condition and dispersal have not produced consistent patterns. Some studies have observed a negative relationship between condition and dispersal. For example, dispersing red-cockaded woodpeckers (*Picoides borealis*) had lower body mass than philopatric individuals (Pasinelli and Walters 2002). However, more studies have observed a positive relationship between condition and dispersal. For example, O'Riain et al. (1996) demonstrated that in naked mole rats (*Heterocephalus glaber*), dispersers are heavier and have higher fat levels than philopatric individuals. Similarly, heavier roe deer (*Capreolus capreolus*) are more likely to disperse and travel further than light individuals (Debeffe et al. 2012), and dispersing ants (*Formica truncorum*) are larger and have greater fat and glycogen content than philopatric ones (Sundstrom 1995).

The existing theoretical literature on condition-dependent dispersal has also come to conflicting conclusions. Negative condition-dependent dispersal may arise as a response to poor or declining habitat conditions (Matthysen 2012) or to competitive pressure from higher condition individuals (the ideal despotic distribution hypothesis; Fretwell 1972). Theoretically, positive associations between body condition and dispersal can arise from environmental stochasticity (Bonte and de la Pena 2009), or kin selection (Gyllenberg et al. 2008, 2011). However, the conditions under which kin selection would produce this association are highly restrictive and unlikely to be applicable to systems with large population sizes and/or with low average relatedness within populations (Gyllenberg et al. 2008, 2011).

The inconsistency in the results of empirical studies of condition-dependent dispersal may be caused by variation among systems in the mechanism driving the relationship between condition and dispersal (e.g., ideal despot vs. kin selection). However, an alternative explanation is that unrecognized interactions between condition and other proximate factors influencing dispersal are producing variation among studies (Cote and Clobert 2012). Perception of, or response to, cues influencing dispersal can be modified by an individual's internal state (Ims and Hjermann 2001; Bowler and Benton 2005). Substantial evidence indicates that behavioral responses to predation risk interact with body condition. For example, Kohler and McPeek (1989) demonstrated that satiated mayflies (*Baetis tricaudatus*) spend more time in refuge in the presence of predators than hungry mayflies. Interactions between perceived environmental conditions and internal state may provide a general explanation for the inconsistency in the results of condition-dependent dispersal studies.

In this study, we asked whether body condition influences dispersal, and whether there is an interaction between predation risk and body condition which shapes dispersal behavior. We tested these questions using backswimmers (*Notonecta undulata),* which are flight-capable, semi-aquatic insects. We manipulated body condition by imposing diet treatments of varying food levels on wild-caught adult backswimmers. Manipulating diet in adults eliminates any possible correlations between body condition and other variables such as body size and maternal condition. However, we note that this manipulation produces a correlation between body condition and prior experience of food availability. To connect dispersal propensity to physiological mechanisms, we analyzed the body composition of a sample of the backswimmers, comparing aspects of body condition tightly linked to physiology (fat and protein content) in backswimmers from across these diet treatments. Finally, we measured the consequences of both condition and predation risk for dispersal in a field mesocosm experiment.

**Figure 1:**
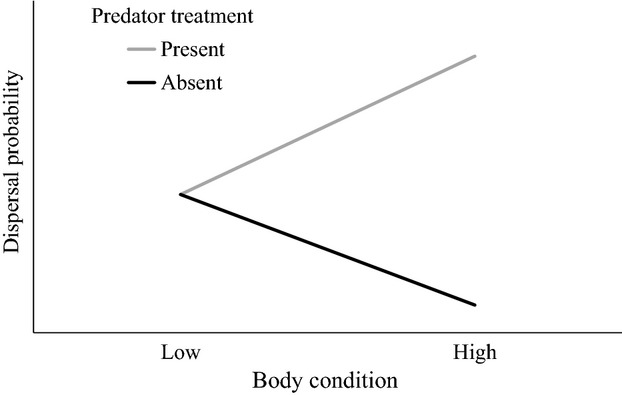
Predicted relationship between dispersal and body condition in predator absent and predator present treatments. In both predator absent and predator present treatments, we predict that low-condition individuals should have high motivation to disperse, but some fraction will not have sufficient capability to disperse. High-condition individuals have high dispersal capability, with low dispersal motivation in the predator absent treatment, and with high dispersal motivation in the predator present treatment.

We hypothesized that in the absence of predation risk, low-condition individuals should have higher dispersal “motivation” than high-condition individuals because their internal state may serve as an indicator that their habitat is of low quality (Clobert et al. 2001; Bowler and Benton 2005). In the predator-absent treatment, this would result in a negative relationship between body condition and dispersal (Fig.1). We have previously demonstrated that *Notonecta* substantially increases dispersal rates in response to the threat of fish predation (McCauley and Rowe 2010; Baines et al. 2014); however, dispersal rates never reach 100%. We hypothesize that the individuals that do not disperse in response to high predation risk are those that are not capable of successfully moving to new habitat patches. As dispersal capability should be positively correlated with body condition (Cockbain 1961), we predicted that high-condition individuals would have the greatest ability to respond to predation risk, and therefore, the relationship between body condition and dispersal should be positive in the predator-present treatment (Fig.1).

## Materials and Methods

### Study system

*Notonecta spp*. (Heteroptera: Notonectidae) are semi-aquatic insects that live in freshwater ponds, streams, and lakes (Clark 1928). They complete their entire life cycle in the aquatic environment, but can disperse by flight among ponds (Clark 1928).

*Notonecta undulata* is generally associated with fishless ponds, but can co-occur with fish (Bendell 1986; Bennett and Streams 1986), including the pumpkinseed sunfish, *Lepomis gibbosus*, which was used as the predator in this experiment. *Lepomis gibbosus* readily consumes *N. undulata* adults in the laboratory (Cook and Streams 1984). In a previous study, McCauley and Rowe (2010) demonstrated that dispersal in *N. undulata* is induced by perceived predation risk from caged *L. gibbosus*. It is not known how body condition influences dispersal in this species.

### Creating variance in notonectid condition

On July 30, 2013, we collected ∽400 adult *N. undulata* from a small fishless pond (∽1300 m^2^) at the Koffler Scientific Reserve (KSR) in Ontario, Canada. On the same day as collection, the notonectids were transported to a laboratory at the University of Toronto in buckets of pond water at densities of ∽50 individuals per 5 L. They were held in these buckets until they were processed in the subsequent 1–2 days.

Notonectids were randomly assigned to one of three diet treatments (low, medium, or high). The hemelytra of all individuals were marked with a unique 3-digit ID number and a symbol to denote their diet treatment, using Sharpie permanent markers. They were then placed individually in plastic drinking cups (diameter: 11 cm, height: 9 cm) filled with ∽250 mL of dechlorinated tap water. Each cup contained a strip of craft foam weighted with a stone, to provide habitat structure. Cups were then placed in a growth chamber set to 24°C, and a 15H day: 9H night cycle to mimic conditions experienced in June in the area where the source pond is located. Individuals of the three diet treatments were positioned randomly within the growth chamber.

Notonectids in the low, medium, and high diet treatments were fed 3, 6, and 12 fruit flies (*Drosophila melanogaster*) per day, respectively. In addition, each notonectid was fed half a cricket twice per week. The diet manipulation began when the notonectids were placed in the growth chamber, and ended on August 19, 2013.

On August 19th, immediately after the diet manipulation ended, we selected a random sample of 11–12 notonectids from each diet treatment and preserved them individually in vials filled with 70% ethanol. These samples were collected so that we could estimate the effects of the diet manipulation on fat mass and protein mass. The remaining notonectids from the diet manipulation were transported to KSR and used in a field experiment measuring dispersal rates from August 19 to September 7, 2013.

### Estimating dispersal rates in the field

In June 2013, we placed an array of ten cattle tanks (tanks: 378 L; 1.35 m × 0.79 m × 0.64 m) in an open field at KSR. We filled the tanks with water and ∽10 rabbit chow pellets as a nutrient base, and left them uncovered to aerate. On July 30th, 2013, we added an equal amount of zooplankton to each cattle tank to provide food for the notonectids.

A fish cage was placed in each cattle tank. Fish cages consisted of a 5-L plastic basket with holes for water exchange and a Styrofoam lid, and were covered in 1 mm mesh screening. These cages allow notonectids to receive visual and olfactory cues signaling the presence of a predator without being consumed. Empty cages were placed in predator absent treatment tanks, to control for the presence of this structure. On August 15th, we caught five pumpkinseed sunfish (*Lepomis gibbosus*; standard length of fish ± standard deviation = 17.5 ± 0.7 cm), and put them in cages in five of the cattle tanks. We fed each fish one cube of frozen bloodworms plus four live notonectids per day. All protocols and procedures related to fish were reviewed and approved by the University of Toronto Bioscience Local Animal Care Committee.

We placed strips of craft foam weighted with stones in all tanks to provide habitat structure, and covered about one-third of each tank with a piece of 70% shade cloth. The shade cloth kept the water temperature of the tanks cool, but did not prevent the notonectids from dispersing.

On August 19th, notonectids were transported from the growth chamber at the University of Toronto and placed in the ten cattle tanks in the field at KSR. Each tank received 12–13 individuals of all three diet treatments, with each tank receiving 36–37 notonectids in total. This notonectid density falls within the natural range for this species (Bennett and Streams 1986).

The cattle tanks were left uncovered for 19 days to allow notonectids to disperse. We measured emigration by recording the markings of each individual present in each tank every 3 days. When dead notonectids were found, we recorded their markings and discarded them away from the cattle tank array. Even notonectids which died as a result of cannibalism could be accounted for in this way, because notonectids consume the insides of their prey and leave the exoskeleton intact, so the ID markings of any cannibalized individuals could be recorded. All individuals that left their original tanks were considered dispersers. All individuals that remained in their original tanks over the entire course of the experiment were considered residents.

Recording the individuals present in all tanks was time consuming and could not be done in a single day. Therefore, the tanks were divided into two time blocks, and we recorded the individuals present within each block every 3 days. Time blocks 1 and 2 contained 4 and 6 tanks, respectively. Both predator treatments were equally represented in both blocks.

At the end of the experiment, we collected all of the “resident” notonectids remaining in the cattle tanks and preserved them individually in vials filled with 70% ethanol. These notonectids were preserved so that we could estimate the residual effects of the diet manipulation on body composition after the field experiment.

### Body composition analysis

We performed body composition analysis on notonectids to test whether diet influenced fat and protein content in these animals. We analyzed all of the notonectids that were preserved immediately after the diet manipulation. We also analyzed a random sample of the notonectids that were classified as residents from the field experiment. In total, 154 individuals were selected for body composition analysis, with a roughly equal number of individuals from each diet treatment.

To analyze body composition, we first identified the sex of each individual. Then, we removed the head, legs, and wings, leaving only the thorax and abdomen. This was done because a pilot project showed that these body parts break off and are lost during the drying process. Removing these body parts is unlikely to alter measurements of body composition because they contain negligible amounts of triglyceride fat and muscle protein. We also chose a random sample of notonectids and dissected their thoraces to determine whether they had developed flight muscles. Dissected individuals were classified as having or lacking developed flight muscles.

The notonectids were then placed in a drying oven at 60°C and dehydrated to a constant weight (approximately 46–48 h). When they were completely dry, we removed them from the drying oven and measured their total dry mass to the nearest 0.01 mg with a Mettler Toledo XS105 scale.

We used chloroform redux to measure the fat mass of each notonectid as per Marden (1989). Individuals were placed in a fat-free thimble (Advantec; 33 mm D x 80 mm L), with 10–11 individuals divided by pieces of filter paper in each thimble. Thimbles were then put in a Soxhlet extractor for 6 hours. This process submerges the specimens in cycles of warm, liquid chloroform to dissolve triglyceride fat and move it away from the specimens. After 6 hours, the specimens were removed from the Soxhlet extractor and were again dehydrated and weighed. Dry fat mass was estimated as the difference between the dry mass and the dry fatless mass.

To measure the protein mass of each notonectid, the dry, fatless notonectids were submerged in 0.2 mol/L potassium hydroxide (KOH) for 48 h (Plaistow and Siva-Jothy 1996). The KOH solution dissolves protein and leaves the exoskeleton intact. We dehydrated and weighed them a final time. The dry protein mass was estimated as the difference between the dry, fatless mass, and the dry, fatless mass after the KOH treatment.

### Statistical analysis

We tested the effect of diet on body composition of individuals preserved immediately after the diet manipulation (“pre” samples) and individuals preserved after the field experiment (“post” samples). The effect of diet treatment on dry fat and dry protein mass of the “pre” samples was analyzed using analysis of covariance, using total dry body mass as a covariate. The effect of diet treatment on dry fat and dry protein mass of the “post” samples was analyzed using a general linear mixed model, with diet treatment and dry body mass as fixed effects, and the identity of the tank from the field experiment as a random effect. The distribution of dry fat mass of the “post” samples was not normally distributed. Therefore, this variable was square-root transformed before analysis of covariance. These analyses were performed in JMP v.11.0.0. (SAS Institute Inc. Cary, NC, USA).

The effects of diet treatment, predator treatment, and time on dispersal rates were analyzed using survival analysis as per Allison (2010). We used a generalized linear mixed model with a binomial error distribution and a logit link, using dispersal status (dispersed or resident) as the response. We allowed dispersal status to be right-censored, to account for individuals that died during the course of the experiment. The fixed effects were time, predator treatment, diet treatment, and all possible interactions. We included tank nested within block as a random effect. This analysis was performed in R v.2.14.2. (R Foundation for Statistical Computing, Vienna, Austria).

## Results

### Fat and protein content in notonectids

**Figure 2:**
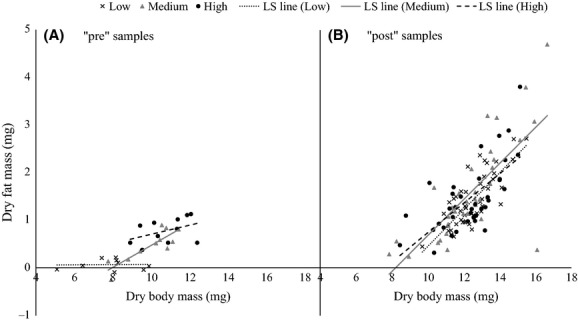
Dry fat mass vs. dry body mass of all three diet treatments. “pre” = notonectids preserved immediately after the diet manipulation (*n* = 34). “post” = notonectids preserved after the field experiment (*n* = 119). Trend lines are least-squares (LS) regression lines. Note that negative values are the result of measurement error associated with the chloroform redux process. Negative values correspond to individuals that have zero or nearly zero levels of triglyceride fat.

Among notonectids preserved immediately after the diet manipulation, fat content depended on diet treatment (diet: *F*_2,28_ = 10.40, *P* = 0.0004; Fig.2A). Independent contrasts showed that individuals from the low diet treatment had the lowest fat content (Medium diet – Low diet: *t*_1_ = 2.40, *P* = 0.0232), and individuals from the high diet treatment had the highest fat content (High diet – Medium diet: *t*_1_ = 2.83, *P* = 0.0085). In addition, the slope of the regression of fat mass on body mass depended on diet treatment (body mass × diet: *F*_2,28_ = 5.73, *P* = 0.0082; Fig.2A). The low diet treatment had the shallowest slope (Medium diet – Low diet: *t*_1_ = 3.37, *P* = 0.0022), but the difference between the slopes of the medium and high diet treatments only approached significance (High diet – Medium diet: *t*_1_ = −1.94, *P* = 0.0630). After the field experiment, notonectids had greater fat mass, on average, than notonectids preserved immediately after the diet manipulation (*F*_1,141_ = 7.12, *P* = 0.0085; Fig.2). This increase was most pronounced in the low diet treatment, so that after the field experiment, there was no longer a discernible effect of diet treatment on fat mass (diet: *F*_2,110_ = 0.50, *P* = 0.6092; Fig.2B). After the field experiment, fat mass depended strongly on body mass (body mass: *F*_1,97.15_ = 34.58, *P* < 0.0001; Fig.2B).

**Figure 3:**
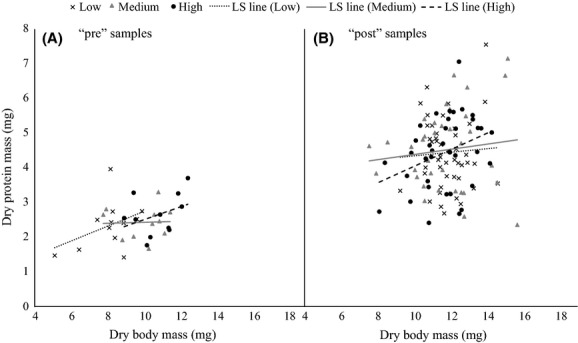
Dry protein mass vs. dry body mass of all three diet treatments. “pre”  = notonectids preserved immediately after the diet manipulation (*n* = 35). “post” = notonectids preserved after the field experiment (*n* = 119). Trend lines are least-squares (LS) regression lines.

All of the notonectids dissected had developed flight muscles (data not shown). Protein mass did not depend on body mass, diet treatment, or their interaction, neither immediately after the diet manipulation (body mass: *F*_1,29_ = 2.56, *P* = 0.1206; diet: *F*_2,29_ = 0.41, *P* = 0.6667; body mass × diet: *F*_2,29_ = 1.07, *P* = 0.3561; Fig.3A), nor after the field experiment (body mass: *F*_1,109.6_ = 0.25, *P* = 0.6163; diet: *F*_2,106.3_ = 0.01, *P* = 0.9867; body mass × diet: *F*_2,107.8_ = 0.61, *P* = 0.5443; Fig.3B). After the field experiment, notonectids had greater protein mass, on average, than notonectids preserved immediately after the diet manipulation (*F*_1,147_ = 59.02, *P* < 0.0001; Fig.3).

There were minor effects of sex and fish treatment on body composition, but these factors did not influence our main conclusions about how the diet treatment affected body composition. These results are presented in the supplementary materials (Fig. S1–S6, Tables S1–S4).

### Dispersal rates in the field

**Figure 4:**
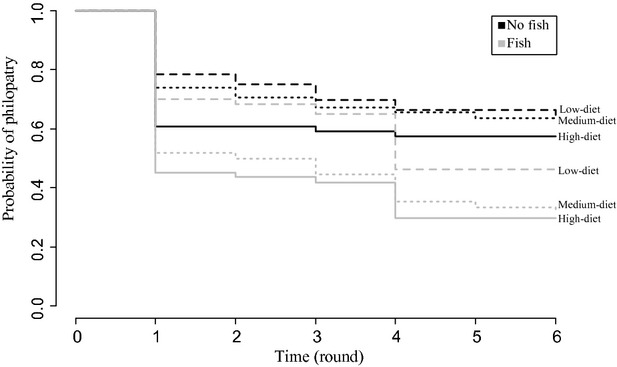
Kaplan–Meier curves for the mean probability of philopatry for each fish × diet treatment in each round. Rounds are separated by 3 days.

Initially, high-condition individuals dispersed more than low-condition individuals, but this difference decreased through time (Fig.4, Fig. S8). Consequently, there was a significant interaction between diet and time (diet × time: 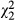 = 11.11, *P* = 0.004; Fig.4) and the main effect of diet was only marginally significant (diet: 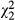 = 5.64, p = 0.060).

More notonectids emigrated from fish tanks than fishless tanks, overall (predator: 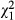 = 23.44, *P* = 1.3 × 10^−6^; Fig.4, Fig. S8), and this difference increased through time (predator × time: 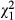 = 4.00, *P* = 0.046; Fig.4). The interactive effect between predator and diet treatments was not significant at any time point (predator × diet: 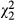 = 2.65, *P* = 0.266; predator × diet × time: 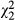 = 3.15, *P* = 0.2066; Fig.4). There was no evidence that sex influenced dispersal probability (Fig. S7).

## Discussion

### The effects of diet on fat and protein stores

We found a positive relationship between fat content and diet treatment, which is consistent with previous studies (Rolff et al. 2004; Dmitriew et al. 2009). As fat is the fuel used for flight in many insects, including *Notonecta* (Gade et al. 2004), this result suggests that dispersal capacity is an increasing function of diet treatment. In addition to fat content depending on diet treatment directly, the slope of the line regressing fat mass onto body mass also depended on diet treatment. This suggests that diet treatment changed patterns of energy use and allocation. Individuals from the low diet treatment allocated extremely low levels of energy to fat, regardless of their structural body size. Conversely, individuals in the medium and high diet treatments had a high enough energy intake to be able to allocate energy to fat stores. These results indicate that individuals from the low diet treatment incur the highest marginal costs of dispersal; the energetic costs of dispersal would be a very high proportion of their total energy stores, compared to individuals from the medium and high diet treatments.

In contrast to our result, previous studies have demonstrated a positive association between body mass and protein mass (e.g., Dmitriew et al. 2009). The lack of a relationship in this experiment could be the result of low detection power; there was especially high variation in protein mass among individuals preserved after the field experiment (Fig.3). This could have been the result of differences in foraging activity or prey selection among individuals. The fact that protein mass did not depend on diet treatment, but was greater in notonectids preserved after the field experiment likely indicates that the food provided to notonectids in the laboratory was low in quality or quantity. However, the variation we observed in dispersal capacity in this experiment is unlikely to be related to variation in protein content affecting flight muscle mass, as protein content was not related to diet treatment, which did have an effect on dispersal. Finally, all notonectids tested had developed flight muscles, suggesting that all notonectids had sufficient protein available to make them flight capable.

### The effects of diet and predators on dispersal

In this study, we found that emigration was an increasing function of condition. Positive condition-dependent dispersal has been demonstrated in a variety of taxa (mostly mammals: O'Riain et al. 1996; Debeffe et al. 2012; and other vertebrates: Pasinelli and Walters 2002). Positive condition-dependent dispersal likely has important consequences for populations. High-condition dispersers travel further distances (Ferrer 1993; Debeffe et al. 2012), compete more successfully for entry into high-quality habitat (Clarke et al. 2008; Bonte et al. 2011), and have greater fecundity (Bonte et al. 2011) than low-condition dispersers. Thus, dispersal that is nonrandom with respect to body condition has consequences for a variety of ecological and evolutionary processes, even when there are no genetic differences between low and high-condition individuals (Edelaar and Bolnick 2012). High-condition dispersers will have a greater impact on the populations that receive them than expected given the number of emigrants and assuming dispersers are a random sample of the population (Benard and McCauley 2008). As a result, fewer immigrants may be required to “rescue” declining populations and restore population growth rates (Brown and Kodric-Brown 1977). Similarly, the same volume of gene flow can be accomplished with a few high-condition dispersers as with a larger number of dispersers of random condition. Therefore, fewer dispersers may be required to alter genetic structure and change rates of local adaptation (Benard and McCauley 2008).

One caveat to the result that dispersal was a positive function of body condition is that individuals from the three diet treatments differed not only in body condition (as measured by energy stores), but also in prior experience of resource availability. Individuals from all diet treatments experienced an increase in resource availability when they were moved to the field experiment (individuals preserved after the field experiment had greater fat and protein mass than individuals preserved immediately after the diet manipulation). If prior experience influences dispersal propensity, then all notonectids should have been motivated to remain in the high-quality mesocosm environments. It is true that individuals from the low diet treatment experienced the largest improvement in habitat quality when they were introduced to the mesocosms, and so may have had the greatest motivation to stay. Therefore, we cannot rule out the possibility that prior experience was contributing to the observed differences in dispersal. However, we argue that in this case, prior experience was likely not the sole driver of the observed pattern. Little is known about the internal and external cues that individuals use to make dispersal decisions, but it is likely that organisms use multiple cues to assess dispersal ability as well as fitness prospects in the current patch vs. a new patch (Clobert et al. 2012). The potential for interactive effects of body condition, prior experience, and current resource availability on dispersal is a fruitful area for study.

The observed relationship between body condition and dispersal has been inconsistent in the empirical literature, but the reasons for this are unknown (Ims and Hjermann 2001; Bowler and Benton 2005). In this study, we tested whether an interaction between predation risk and body condition contributed to this inconsistency, but did not find support for this hypothesis. The relationship between condition and dispersal was similar in both predator treatments; individuals from the high diet treatment had the highest emigration rates, and individuals from the low diet treatment had the lowest emigration rates, regardless of predator treatment. This result does not match our prediction for how condition-dependent dispersal should be altered by predation risk. However, further tests of this interaction in a greater diversity of systems may provide other insights.

In this study, we observed a decrease through time in the magnitude of the effect of diet on emigration. This change through time likely arose from the common diet of all individuals in the cattle tank mesocosms. During this portion of the experiment, individuals from all three diet treatments were in the same tanks and had access to the same resources. Differences between diet treatments in fat mass decreased over the course of the field experiment, and overall, fat and protein mass increased relative to levels at the start of the field experiment. This suggests that, in these mesocosms, notonectids from the low and medium diet treatments could compensate for their earlier diet restriction, reaching body conditions equivalent to those of the individuals from the high diet treatment. It appears that as notonectids from the low and medium treatment improved their body condition, their dispersal levels also increased, eliminating the differences between their dispersal rates and those of notonectids from the high food treatment that entered the experiment in good condition and with sufficient fat to fuel dispersal.

As expected, we observed that *N. undulata* increases dispersal in response to predation risk. This is consistent with previous studies of the effect of predation risk on dispersal, including similar studies conducted on *N. undulata* (McCauley and Rowe 2010; Baines et al. 2014). This result is predicted by general theory stating that individuals should maximize their fitness by moving away from poor-quality or dangerous patches in favor of high-quality, safe patches, as long as the benefits of leaving outweigh the costs of dispersal (Bonte et al. 2012; Clobert et al. 2012). Poethke et al. (2010) specifically investigated the evolution of predator induced dispersal and their model demonstrated that prey should evolve higher dispersal rates in the presence of predators when temporal autocorrelation in predation risk is high, as it is in the *Notonecta* system. This dispersal response provides spatial refuge for prey, and can enable regional coexistence of predators and prey, even when temporal autocorrelation in predation risk is not perfect (Hanski 2001).

Condition-dependent dispersal will likely have strong effects on structure, stability, and evolution in metapopulations. Given this, it is important to understand the mechanisms by which condition influences dispersal, and how individual condition may interact with the ecological or social context individuals experience to shape dispersal behavior. Research on these interactions is likely to provide insights into why previous work has observed inconsistencies in the association between individual condition and dispersal behavior. Future research investigating the relationship between condition and dispersal, and testing whether the direction of this relationship is context-dependent, will provide results which have important implications for the ecology and evolution of metapopulations.

## Supporting Information

**Figure S1.** Dry fat mass vs. dry body mass ± 95% confidence of fit bands for males and females in all diet treatments among individuals preserved immediately after the diet manipulation (“pre”; *n* = 34). **Figure S2.** Dry fat mass vs. dry body mass ± 95% confidence of fit bands for males and females in all diet treatments and both fish treatments. **Table S1.** Results of GLMM modeling the effects of diet treatment, fish treatment, sex, dry body mass, and all possible interactions on the square root of dry fat mass of the “post” samples. **Figure S3**. Dry protein mass vs. dry body mass ± 95% confidence of fit bands for males and females in all diet treatments among individuals preserved immediately after the diet manipulation (“pre”; *n* = 35). **Figure S4.** Dry protein mass vs. dry body mass ± 95% confidence of fit bands for males and females in all diet treatments and both fish treatments. **Table S2.** Results of GLMM modeling the effects of diet treatment, fish treatment, sex, dry body mass, and all possible interactions on dry protein mass of the “post” samples. **Figure S5.** Dry fat mass vs. dry body mass ± 95% confidence of fit bands for males and females preserved immediately after the diet manipulation (“pre”; *n* = 34) and after the field experiment (“post”; *n* = 119). **Figure S6**. Dry protein mass vs. dry body mass ± 95% confidence of fit bands for males and females preserved immediately after the diet manipulation (“pre”; *n* = 35) and after the field experiment (“post”; *n* = 119). **Table S4.** Results of ANCOVA modeling the effects of timing of preservation (type), dry body mass, sex, and all possible interactions on dry protein mass. **Figure S7.** The percent of all experimental animals of each sex in the “pre” (notonectids preserved immediately after the diet manipulation (*n* = 34)) and “post” (notonectids preserved after the field experiment (*n* = 119)) samples. **Figure S8.** (A) Mean proportion of philopatric individuals for each fish × diet treatment in the first 3 days of the field experiment. (B) Mean proportion of philopatric individuals for each fish × diet treatment in the last 3 days of the experiment.
