# Supplementary material for: Dispersal depends on body condition and predation risk in the semi-aquatic insect, Notonecta undulata
Source: Ecol Evol. 2015 May 20;5(12):2307–16. doi: 10.1002/ece3.1508 (PMC4475364; doi:10.1002/ece3.1508)
Supplement: Supplementary file 1 — Figure S1. Dry fat mass vs. dry body mass ± 95% confidence of fit bands for males and females in all diet treatments among individuals preserved immediately after the diet manipulation (“pre”; n = 34). Figure S2. Dry fat mass vs. dry body mass ± 95% confidence of fit bands for males and females in all diet treatments and both fish treatments. Table S1. Results of GLMM modeling the effects of diet treatment, fish treatment, sex, dry body mass, and all possible interactions on the square root of dry fat mass of the “post” samples. Figure S3. Dry protein mass vs. dry body mass ± 95% confidence of fit bands for males and females in all diet treatments among individuals preserved immediately after the diet manipulation (“pre”; n = 35). Figure S4. Dry protein mass vs. dry body mass ± 95% confidence of fit bands for males and females in all diet treatments and both fish treatments. Table S2. Results of GLMM modeling the effects of diet treatment, fish treatment, sex, dry body mass, and all possible interactions on dry protein mass of the “post” samples. Figure S5. Dry fat mass vs. dry body mass ± 95% confidence of fit bands for males and females preserved immediately after the diet manipulation (“pre”; n = 34) and after the field experiment (“post”; n = 119). Figure S6. Dry protein mass vs. dry body mass ± 95% confidence of fit bands for males and females preserved immediately after the diet manipulation (“pre”; n = 35) and after the field experiment (“post”; n = 119). Table S4. Results of ANCOVA modeling the effects of timing of preservation (type), dry body mass, sex, and all possible interactions on dry protein mass. Figure S7. The percent of all experimental animals of each sex in the “pre” (notonectids preserved immediately after the diet manipulation (n = 34)) and “post” (notonectids preserved after the field experiment (n = 119)) samples. Figure S8. (A) Mean proportion of philopatric individuals for each fish × diet treatment in the first 3 days of the field experim [file ece30005-2307-sd1.docx]

**Dispersal depends on body condition and predation risk in the semi-aquatic insect, *Notonecta undulata***

**Celina B. Baines, Shannon J. McCauley, & Locke Rowe**

**Supplementary Materials**

1. **The effects of sex and fish treatment on body composition**

**Methods**

*Statistical analysis*

Analysis of covariance was used to test the effects of diet, sex, and dry body mass on dry fat mass and dry protein mass of the “pre” samples. There was insufficient replication to test the effect of the interactions between sex and the other two variables (diet and dry body mass); we therefore excluded these interaction terms from the ANCOVA model, so that the final models were dry fat (protein) mass ~ diet + dry body mass + diet × dry body mass + sex.

To analyze dry fat mass and dry protein mass of individuals preserved after the field experiment (“post” samples), we used general linear mixed models with diet treatment, fish treatment, sex, dry body mass, and all possible interactions as fixed factors, and tank ID as a random factor. The distribution of dry fat mass of the “post” samples was not normally distributed. Therefore, this variable was square-root transformed before analysis.

**Results**

*Dry fat mass*

Among notonectids preserved immediately after the diet manipulation, fat content depended on diet treatment (diet: F_2,27_ = 10.00, p = 0.0006; Figure S1), and the interaction between diet and dry body mass (body mass × diet: F_2,27_ = 5.27, p = 0.01). There was no effect of sex on dry fat mass (sex: F_1,27_ = 0.02, p = 0.90; Figure S1).


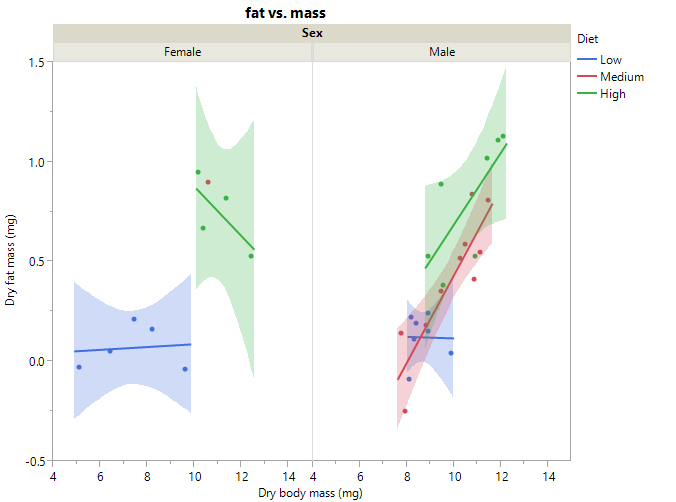


**Figure S1.** Dry fat mass vs dry body mass +/- 95% confidence of fit bands for males and females in all diet treatments among individuals preserved immediately after the diet manipulation (“pre”; n = 34).

Among notonectids preserved after the field experiment, dry fat mass was strongly correlated with dry body mass (Figure S2, Table S1). Females had slightly greater dry fat mass than males with the same dry body mass (Figure S2, Table S1). Neither diet treatment nor fish treatment had a significant effect on dry fat mass (Table S1).


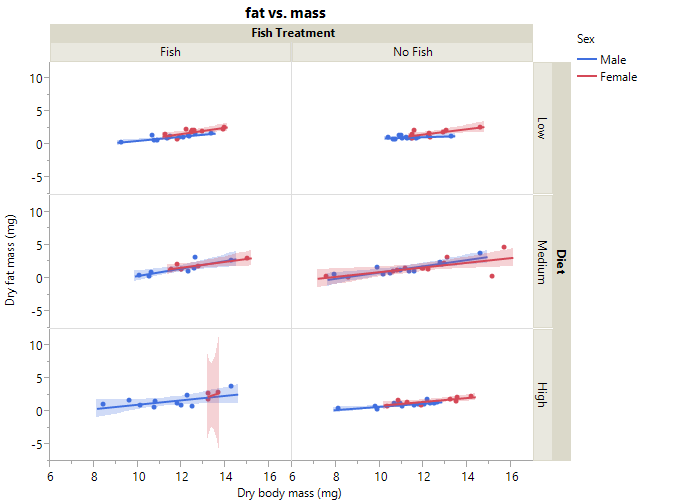


**Figure S2.** Dry fat mass vs dry body mass +/- 95% confidence of fit bands for males and females in all diet treatments and both fish treatments. These individuals were preserved after the field experiment (“post”; n = 119).

**Table S1.** Results of GLMM modeling the effects of diet treatment, fish treatment, sex, dry body mass, and all possible interactions on the square root of dry fat mass of the “post” samples. * indicates significance at α = 0.05.

| Source | DFNum | DFDen | F | P |
| --- | --- | --- | --- | --- |
| Dry body mass | 1 | 93.34 | 13.35 | 0.0004* |
| Sex | 1 | 93.70 | 4.05 | 0.0470* |
| Dry body mass : Sex | 1 | 92.77 | 0.57 | 0.4503 |
| Diet | 2 | 91.81 | 0.54 | 0.5818 |
| Dry body mass : Diet | 2 | 92.34 | 0.42 | 0.6576 |
| Sex : Diet | 2 | 91.86 | 0.77 | 0.4681 |
| Dry body mass : Sex: Diet | 2 | 92.64 | 1.11 | 0.3355 |
| Fish | 1 | 43.67 | 0.01 | 0.9204 |
| Dry body mass : Fish | 1 | 93.34 | 0.89 | 0.3480 |
| Sex : Fish | 1 | 93.70 | 0.01 | 0.9156 |
| Dry body mass : Sex: Fish | 1 | 92.77 | 0.24 | 0.6268 |
| Diet : Fish | 2 | 91.81 | 0.10 | 0.9057 |
| Dry body mass : Diet : Fish | 2 | 92.34 | 0.09 | 0.9181 |
| Sex : Diet : Fish | 2 | 91.86 | 0.52 | 0.5953 |
| Dry body mass : Sex : Diet : Fish | 2 | 92.64 | 0.16 | 0.8532 |

*Dry protein mass*

Among notonectids preserved immediately after the diet manipulation, protein content did not depend on body mass, diet treatment, or the interaction between diet and dry body mass (body mass: F_1,28_ = 1.33, p = 0.26; diet: F_2,28_ = 0.35, p = 0.71; body mass × diet: F_2,28_ = 0.98, p = 0.39; Figure S3). There was no effect of sex on dry protein mass (sex: F_1,28_ = 1.06, p = 0.31; Figure S3).


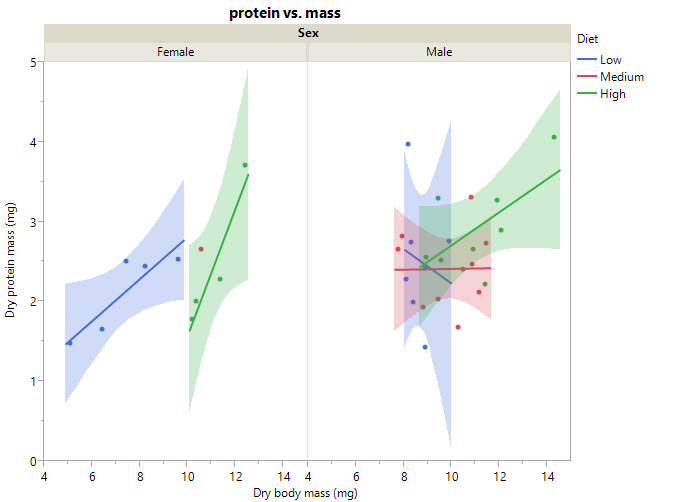


**Figure S3**. Dry protein mass vs dry body mass +/- 95% confidence of fit bands for males and females in all diet treatments among individuals preserved immediately after the diet manipulation (“pre”; n = 35).

Among notonectids preserved after the field experiment, there was a significant interaction between dry body mass and fish treatment on dry protein mass (Figure S4, Table S2); there was a steeper positive slope of dry protein mass on dry body mass in fish tanks than in fishless tanks. There was also a significant interaction between dry body mass and sex (Figure S4, Table S2); the slope of dry protein mass on dry body mass was slightly more positive in males than in females.


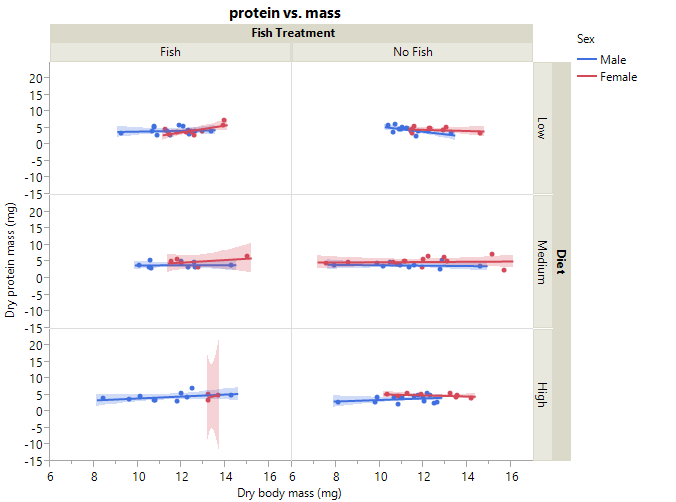


**Figure S4.** Dry protein mass vs dry body mass +/- 95% confidence of fit bands for males and females in all diet treatments and both fish treatments. These individuals were preserved after the field experiment (“post”; n = 119).

**Table S2.** Results of GLMM modeling the effects of diet treatment, fish treatment, sex, dry body mass, and all possible interactions on dry protein mass of the “post” samples. * indicates significance at α = 0.05.

| Source | DFNum | DFDen | F | P |
| --- | --- | --- | --- | --- |
| Dry body mass | 1 | 91.61 | 0.18 | 0.6709 |
| Sex | 1 | 91.47 | 0.06 | 0.8079 |
| Dry body mass: Sex | 1 | 90.89 | 4.62 | 0.0343* |
| Diet | 2 | 91.70 | 0.29 | 0.7502 |
| Dry body mass : Diet | 2 | 92.41 | 0.19 | 0.8270 |
| Sex : Diet | 2 | 92.76 | 1.51 | 0.2258 |
| Dry body mass : Sex : Diet | 2 | 92.13 | 0.57 | 0.5692 |
| Fish | 1 | 28.22 | 1.64 | 0.2108 |
| Dry body mass : Fish | 1 | 91.61 | 8.74 | 0.0040* |
| Sex : Fish | 1 | 91.47 | 3.09 | 0.0821 |
| Dry body mass : Sex : Fish | 1 | 90.89 | 0.52 | 0.4711 |
| Diet : Fish | 2 | 91.70 | 0.38 | 0.6830 |
| Dry body mass : Diet : Fish | 2 | 92.41 | 1.95 | 0.1476 |
| Sex : Diet : Fish | 2 | 92.76 | 0.57 | 0.5685 |
| Dry body mass : Sex : Diet : Fish | 2 | 92.13 | 0.14 | 0.8687 |

1. **The effect of sex on mass gain during the field experiment**

**Methods**

We tested the effect of sex on fat and protein mass gain during the field experiment using ANCOVA. The response variable was dry fat mass or dry protein mass, and the predictor variables were type (i.e. timing of preservation – before or after the field experiment), dry body mass, sex, and all possible interactions.

**Results**

*Dry fat mass*

Notonectids preserved after the field experiment had greater fat mass than those preserved immediately after the diet manipulation (Figure S5, Table S3). There was no effect of sex on fat mass gain (Figure S5, Table S3).

**
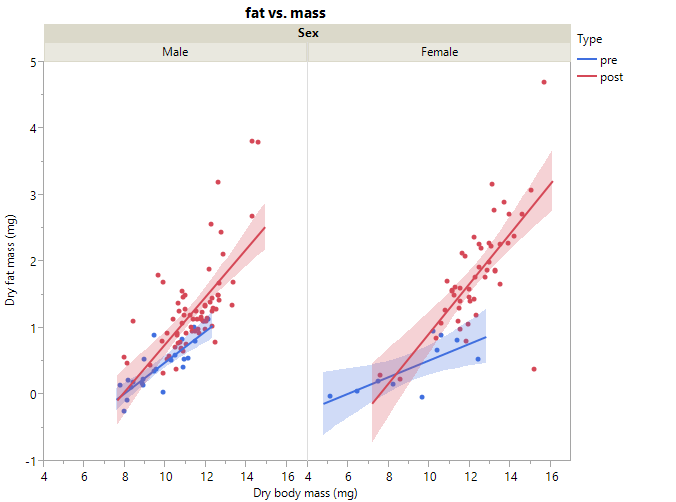
**

**Figure S5.** Dry fat mass vs dry body mass +/- 95% confidence of fit bands for males and females preserved immediately after the diet manipulation (“pre”; n=34) and after the field experiment (“post”; n=119).

**Table S3.** Results of ANCOVA modeling the effects of timing of preservation (type), dry body mass, sex, and all possible interactions on dry fat mass. * indicates significance at α = 0.05.

| Source | DF | F | P |
| --- | --- | --- | --- |
| Type | 1 | 16.96 | <0.0001* |
| Dry body mass | 1 | 79.49 | <0.0001* |
| Type : Dry body mass | 1 | 9.31 | 0.0027* |
| Sex | 1 | 0.07 | 0.7953 |
| Type : Sex | 1 | 1.13 | 0.2886 |
| Dry body mass : Sex | 1 | 0.55 | 0.4589 |
| Type : Dry body mass : Sex | 1 | 1.07 | 0.3023 |

*Dry protein mass*

Notonectids preserved after the field experiment had greater protein mass than those preserved immediately after the diet manipulation (Figure S6, Table S4). There was no effect of sex on protein mass gain (Figure S6, Table S4).


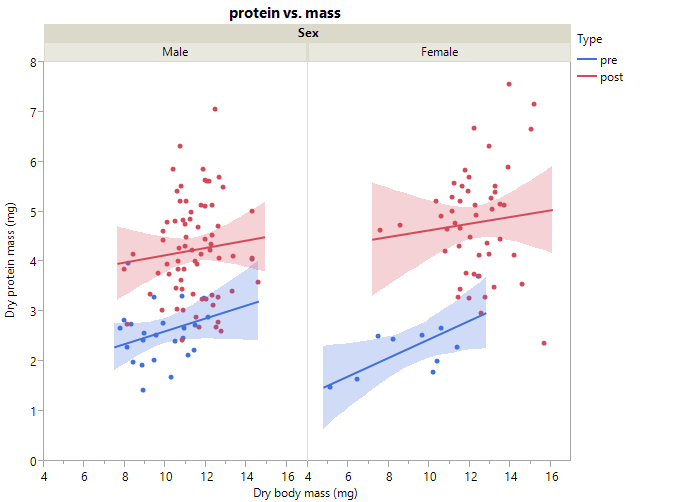


**Figure S6**. Dry protein mass vs dry body mass +/- 95% confidence of fit bands for males and females preserved immediately after the diet manipulation (“pre”; n=35) and after the field experiment (“post”; n=119).

**Table S4.** Results of ANCOVA modeling the effects of timing of preservation (type), dry body mass, sex, and all possible interactions on dry protein mass. * indicates significance at α = 0.05.

| Source | DF | F | P |
| --- | --- | --- | --- |
| Type | 1 | 42.16 | <0.0001* |
| Dry body mass | 1 | 4.05 | 0.0461* |
| Type : Dry body mass | 1 | 0.60 | 0.4406 |
| Sex | 1 | 0.57 | 0.4507 |
| Type : Sex | 1 | 1.16 | 0.2836 |
| Dry body mass : Sex | 1 | 0.05 | 0.8226 |
| Type : Dry body mass : Sex | 1 | 0.08 | 0.7790 |

1. **The effect of sex on dispersal probability**

**Methods**

We tested whether the sex ratio of the “pre” samples was different from the sex ratio of the “post” samples using a χ^2^ contingency test.

**Results**

The sex ratio was strongly male-skewed in both “pre” and “post” samples (Figure S7). There was no significant difference in the sex ratio of “pre” and “post” samples (χ^2^_1_ = 0.9860, p = 0.3207).

**Figure S7.** The percent of all experimental animals of each sex in the “pre” (notonectids preserved immediately after the diet manipulation (n = 34)) and “post” (notonectids preserved after the field experiment (n = 119)) samples.

1. **Initial and final probability of philopatry**


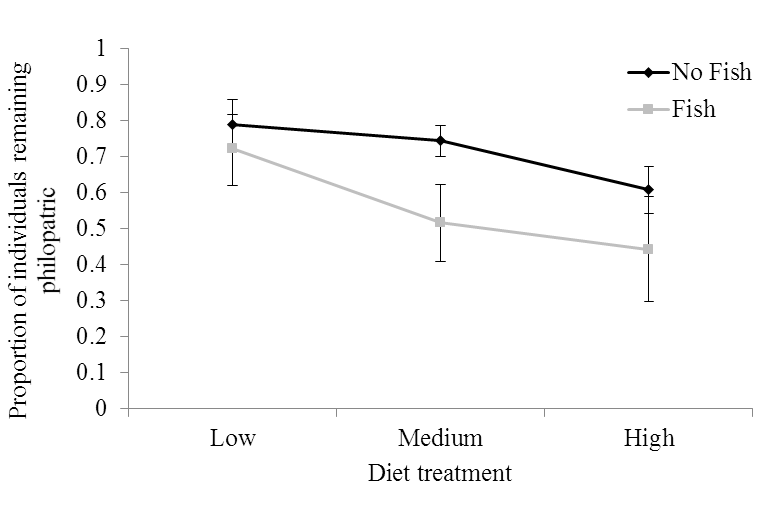

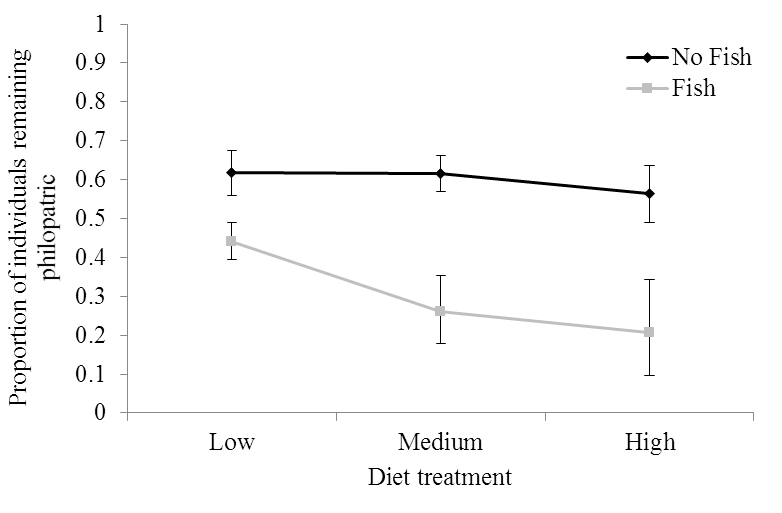


**A**. Initial philopatry

**B**. Final philopatry

**Figure S8.** A) Mean proportion of philopatric individuals for each fish × diet treatment in the first three days of the field experiment. B) Mean proportion of philopatric individuals for each fish × diet treatment in the last three days of the experiment.
